# Beyond Pain Relief: Quality of Life and Functional Outcomes Following Minimally Invasive Excision of Deep Endometriosis

**DOI:** 10.3390/diseases14060216

**Published:** 2026-06-15

**Authors:** Andrei Manu, Elena Poenaru, Arina-Ilinca Gheorghe, Smaranda Stoleru, Alexandra Irma Gabriela Baușic, Bogdan-Cătălin Coroleucă, Ciprian-Andrei Coroleucă, Cristina-Maria Iacob, Mihaela Arina Banu, Anca-Mihaela Hashemi, Maria-Bianca Nițescu, Oana-Miruna Peiu, Elvira Brătilă

**Affiliations:** 1 Doctoral School, “Carol Davila” University of Medicine and Pharmacy, 020021 Bucharest, Romania; andrei.manu@drd.umfcd.ro (A.M.); cristina-maria.iacob@drd.umfcd.ro (C.-M.I.); mihaela-arina.banu@drd.umfcd.ro (M.A.B.); 2 Clinical Hospital of Obstetrics and Gynecology “Prof. Dr. Panait Sîrbu”, 060251 Bucharest, Romania; alexandra.bausic@umfcd.ro (A.I.G.B.); ccoroleuca@yahoo.com (B.-C.C.); anca-mihaela.hashemi@rez.umfcd.ro (A.-M.H.); maria-bianca.nitescu@rez.umfcd.ro (M.-B.N.); oana-miruna.peiu@rez.umfcd.ro (O.-M.P.); elvirabarbulea@gmail.com (E.B.); 3 Faculty of Medicine, “Carol Davila” University of Medicine and Pharmacy, 050474 Bucharest, Romania; arina-ilinca.gheorghe0721@stud.umfcd.ro (A.-I.G.); smaranda.stoleru@umfcd.ro (S.S.)

**Keywords:** endometriosis, deep infiltrating endometriosis, quality of life, minimally invasive surgery, GIQLI, SF-36, bowel resection

## Abstract

Background: Deep infiltrating endometriosis (DIE), particularly when involving the bowel, significantly impairs health-related quality of life (HRQoL) and gastrointestinal function. This study aimed to evaluate the short- and mid-term impact of minimally invasive excision on these parameters in a large multicenter cohort. Methods: A retrospective observational study was conducted on 837 patients treated for endometriosis in two tertiary referral centers between 2018 and 2024. All patients underwent laparoscopic or robotic-assisted excision. Quality of life was assessed preoperatively and at 6 months (VAS: *n* = 69; SF-36: *n* = 100; GIQLI: *n* = 98) and 12 months (VAS: *n* = 30; SF-36: *n* = 46; GIQLI: *n* = 44) postoperatively, using validated patient-reported outcome measures (PROMs): the Visual Analog Scale (VAS) for pain, the Short Form-36 (SF-36) survey, and the Gastrointestinal Quality of Life Index (GIQLI). Results: The study population presented with predominantly advanced disease (Stage III–IV in 83.4% of cases), with 39.7% of patients undergoing segmental bowel resection. Postoperatively, a statistically significant reduction was observed in dysmenorrhea (VAS 7.6 vs. 5.0, *p* < 0.001) and chronic pelvic pain. The SF-36 scores improved significantly across all eight domains at 6 months, with the most dramatic recovery seen in Role Physical (*p* < 0.001) and Bodily Pain (*p* < 0.001). Regarding digestive function, the mean GIQLI score showed a progressive increase, reaching statistical significance at 12 months compared to baseline (112.6 vs. 106.6, *p* = 0.027), indicating superior long-term functional outcomes. Conclusions: Multidisciplinary minimally invasive surgery for deep infiltrating endometriosis was associated with significant and sustained improvements in quality of life among patients with available follow-up. Gastrointestinal quality of life, as measured by GIQLI, improved significantly at 12 months postoperatively, including in patients who underwent segmental bowel resection. Systematic use of PROMs is essential for accurate patient counseling and outcome monitoring.

## 1. Introduction

Endometriosis represents a complex, chronic inflammatory pathology characterized by the presence of endometrial-like tissue outside the uterine cavity, predominantly affecting women of reproductive age. It is a multifaceted disease associated with a broad spectrum of clinical manifestations, ranging from chronic pelvic pain and infertility to severe functional impairment, all of which significantly deteriorate health-related quality of life (HRQoL) [1,2]. The global burden of endometriosis extends beyond physical symptoms, with affected women experiencing profound reductions across all domains of well-being, including vitality, social functioning, and mental health. Multiregional studies have demonstrated that endometriosis impairs both HRQoL and work productivity across diverse countries and ethnicities, with women losing on average 10.8 h of work weekly, mainly owing to reduced effectiveness while working [3]. The chronic nature of the disease and the associated diagnostic delays, averaging 6.7 years from symptom onset to surgical diagnosis, further exacerbate the psychological and social burden experienced by these patients [3,4]. Beyond its macroscopic presentation, recent research has highlighted the molecular heterogeneity of the ectopic endometrium. The specific expression of estrogen (ER) and progesterone receptors (PR), alongside apoptosis-related markers like Bcl-2 and proliferation markers such as Ki-67, dictates the invasive potential and clinical aggressiveness of the disease, necessitating a nuanced understanding of its biological profile [5].

Deep infiltrating endometriosis (DIE), particularly when involving the colorectal compartment, is strongly linked to complex symptom profiles that extend beyond gynecological pain, influencing multiple domains of daily life [6]. Gastrointestinal complaints, such as dyschezia, constipation, diarrhea, and bloating, are highly prevalent in these patients. These symptoms often correlate poorly with the extent of pelvic lesions alone but represent a major contributor to disability and social limitation [7]. Consequently, accurate preoperative assessment is fundamental for planning the appropriate surgical strategy. While Transvaginal Ultrasound (TVUS) serves as the first-line imaging modality, Magnetic Resonance Imaging (MRI) offers complementary diagnostic value, both being essential for the precise mapping of deep lesions and facilitating a tailored multidisciplinary approach [8].

As emphasized in recent reviews, PROMs allow for a structured assessment of the “burden of disease” that clinical examination alone cannot capture, supporting their integration into routine care to better understand the patient’s perspective [9,10,11]. The use of PROMs enables clinicians to quantify the multidimensional impact of the disease, facilitate patient-provider communication, and provide objective metrics for counseling and treatment planning [12,13]. While 48 different PROMs have been identified in the literature for assessing various dimensions of endometriosis impact, only a subset demonstrates adequate disease-specificity, reliability, and responsiveness to change, underscoring the need for careful selection when implementing these tools in clinical practice [13].

Generic instruments, such as the Short Form-36 (SF-36) survey, are commonly utilized because they enable structured, domain-level reporting of recovery after treatment [10]. The SF-36 has been extensively validated in endometriosis populations and demonstrates excellent responsiveness to treatment effects, particularly in physical functioning and bodily pain domains, making it a preferred choice for comparative outcome studies [10,14]. Alongside these, endometriosis-specific PROMs, such as the Endometriosis Health Profile (EHP-30), are widely applied and considered highly sensitive to disease-related limitations [15]. The EHP-30, developed through systematic patient interviews across multiple countries, captures the unique experiential aspects of endometriosis that generic instruments may overlook, including dimensions of control, powerlessness, and disease-specific emotional distress [15,16]. Contemporary guidelines recommend combining disease-specific tools with generic instruments to comprehensively capture both endometriosis-specific and general health impacts, thereby enabling more nuanced clinical interpretation and shared decision-making [11,13]. Recent developments include the creation of targeted instruments such as the Endometriosis Symptom Diary (ESD) and Endometriosis Impact Scale (EIS), which provide standardized, FDA-compliant measures for clinical trial endpoints while maintaining patient-centeredness through extensive qualitative validation [17].

Surgery remains the cornerstone of care for symptomatic endometriosis refractory to medical therapy. Minimally invasive surgery (MIS) has been shown to offer superior outcomes in terms of recovery and cosmetic results, while significantly improving quality of life and fertility rates in patients with DIE [18]. The impact of surgical excision on quality of life has been extensively documented, with systematic reviews and meta-analyses consistently reporting postoperative improvement in HRQoL scores across major domains [19]. Specifically, surgery has been shown to improve physical functioning, bodily pain, vitality, and emotional well-being, with effect sizes ranging from moderate to large depending on the baseline severity and the extent of disease [19,20]. However, the durability of this improvement remains a critical area of investigation. Long-term follow-up is essential to clarify the sustainability of symptom relief and the impact of potential recurrence on HRQoL trajectories [21]. Longitudinal studies tracking patients over extended periods have revealed heterogeneous recovery patterns, with distinct subgroups experiencing different trajectories of pain and quality of life improvement, underscoring the importance of individualized prognostic counseling [22]. Predictive factors for sustained improvement include younger age, absence of deep endometriotic nodules, complete excision of visible lesions, and lower baseline pain intensity [22,23]. Evidence syntheses support the beneficial effect of surgery on pain and QoL outcomes across various phenotypes, yet they also underline the methodological heterogeneity across studies, particularly regarding follow-up intervals and the instruments employed [20].

Functional outcomes beyond pain are particularly relevant in DIE involving the bowel, where gastrointestinal function can significantly influence postoperative recovery and may not always follow the same improvement curve as pain reduction [24]. Surgical technique, particularly the adoption of nerve-sparing and vessel-preserving approaches, plays a critical role in minimizing postoperative gastrointestinal morbidity and preserving autonomic function [25,26]. The choice between conservative approaches (shaving, discoid resection) and more radical segmental resection is determined by lesion characteristics, with emerging evidence from systematic reviews suggesting that while segmental resection achieves superior pain relief, it is associated with higher rates of certain bowel complaints compared to conservative surgical approaches [27,28]. Recent high-quality meta-analyses have quantified these trade-offs, demonstrating that segmental resection provides better dysmenorrhea and dyspareunia outcomes but may result in transient increases in bowel frequency and urgency during the early postoperative period [27]. Patient adherence to long-term follow-up is often limited, which can affect the interpretability of longitudinal data [29]. Therefore, datasets that systematically collect preoperative and postoperative PROMs at defined time points are valuable for mapping domain-specific recovery patterns [30]. At baseline, disease phenotype and symptom dominance remain important determinants of impairment, with chronic pelvic pain consistently associated with the lowest QoL scores [31]. For patients with DIE, the interplay between symptom relief and the preservation of reproductive potential remains a primary concern during surgical counseling; in the Romanian population, multicenter research has highlighted that addressing endometriosis-related health challenges from a young age is crucial for optimizing reproductive outcomes [32].

In cases of bowel endometriosis, functional recovery is shaped by the surgical technique and the emphasis on nerve- and vascular-sparing principles. Cohorts managed with nerve-sparing principles have demonstrated significant postoperative improvement in gastrointestinal function when measured by validated instruments such as the Gastrointestinal Quality of Life Index (GIQLI) [26]. Prospective long-term evaluations have shown progressive improvement in bowel function following surgery, with initial transient deterioration in some parameters resolving by 12 months, ultimately resulting in functional status superior to preoperative baseline [28]. Quality of life meta-analyses focusing specifically on colorectal endometriosis have confirmed robust improvements in both generic (SF-36) and disease-specific (EHP-30) QoL measures at 6 and 12 months postoperatively, with effect sizes most pronounced in the physical health domains [33]. Finally, the role of minimally invasive platforms, including robotic surgery, continues to expand in complex endometriosis procedures, aligning surgical innovation with robust, patient-centered outcomes [34].

In this context, the present study aims to evaluate the short- and mid-term impact of minimally invasive excision for endometriosis on quality of life and gastrointestinal function in a large, multicenter cohort. Specifically, we analyzed the evolution of standardized QoL domains (SF-36) and digestive functional scores (GIQLI) at 6 and 12 months postoperatively, in relation to the extent of surgical resection.

## 2. Materials and Methods

### 2.1. Study Design and Settings

This multicenter retrospective observational study was conducted over a 7-year period, from January 2018 to December 2024. The study population included patients treated in two reference centers for endometriosis surgery in Bucharest, Romania: the Clinical Hospital of Obstetrics and Gynecology “Prof. Dr. Panait Sîrbu” and Memorial Hospital.

The study protocol was approved by the Ethics Committees/Institutional Review Boards. The research was conducted in strict accordance with the ethical standards laid down in the 1964 Declaration of Helsinki and its later amendments. Informed consent regarding the surgical risks and the potential use of medical data for scientific purposes was obtained from all patients prior to surgery.

### 2.2. Patient Selection

**Figure 1:**
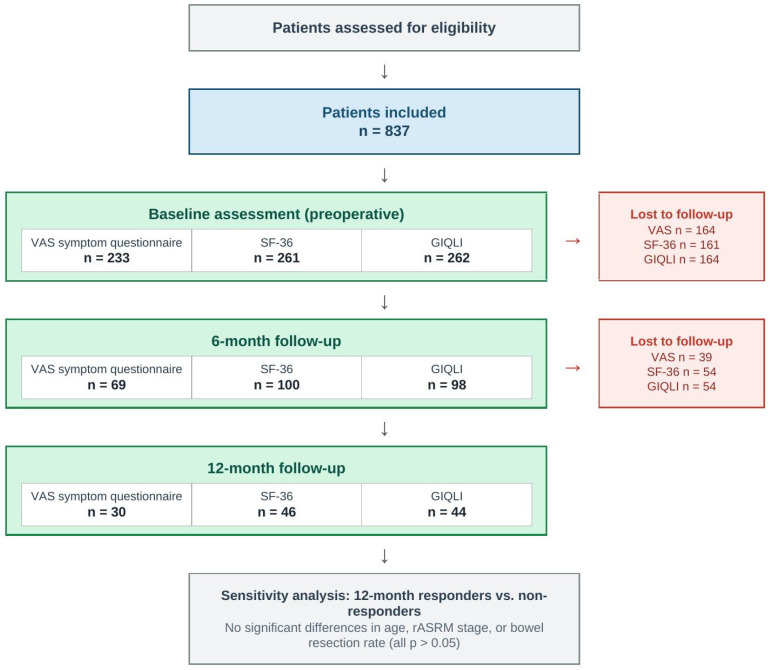
Patient flow diagram showing the number of available responses for each patient-reported outcome measure at each timepoint. Sensitivity analysis at 12 months revealed no significant differences between responders and non-responders regarding age, rASRM.

The study enrolled patients diagnosed with deep infiltrating endometriosis (DIE), characterized as endometriotic lesions extending more than 5 mm below the peritoneal surface, following the current ESHRE classification criteria, who underwent surgical treatment within the specified timeframe. Patient inclusion, available follow-up data for each patient-reported outcome measure, and attrition at each timepoint are summarized in the flow diagram (Figure 1).

- Inclusion criteria: (1) Women of reproductive age (18–45 years); (2) Elective minimally invasive surgery (laparoscopic or robotic) for endometriosis; (3) Histopathological confirmation of the diagnosis; (4) Availability of preoperative and postoperative quality of life data (at least one follow-up point at 6 or 12 months).
- Exclusion criteria: (1) History of pelvic malignancy or borderline ovarian tumors; (2) Pregnancy at the time of surgery; (3) Incomplete medical records; (4) Presence of concurrent inflammatory bowel disease (IBD) or other non-endometriosis colorectal pathologies.

### 2.3. Surgical Management and Postoperative Care

All surgical interventions were performed by a consistent multidisciplinary team comprising senior gynecologists, general surgeons, and urologists with extensive experience in deep infiltrating endometriosis (DIE). The surgical approach was strictly minimally invasive (laparoscopic or robotic-assisted).

The primary objective was the complete macroscopic excision of all endometriotic lesions. For bowel involvement, the technique was tailored to the lesion characteristics, favoring nerve-sparing dissection to preserve autonomic function: Rectal Shaving/Discoid Resection/Segmental Resection followed by end-to-end anastomosis.

In selected cases, postoperative hormonal suppression was administered using GnRH agonists for a duration of 2 to 4 months, in accordance with institutional protocol. No patient received hormonal suppression exceeding 4 months. Following completion of GnRH therapy, patients wishing to conceive were counselled regarding natural conception or assisted reproductive techniques (ART/IVF), while those not seeking pregnancy were maintained on combined oral contraceptives.

### 2.4. Data Collection and Instruments

Clinical and surgical data were retrieved from the institutional electronic databases. Quality of life and symptom severity were assessed preoperatively and postoperatively using three standardized instruments:

- Visual Analog Scale (VAS): Assessing dysmenorrhea, dyspareunia, and chronic pelvic pain (0–10 scale).
- Short Form-36 (SF-36): Evaluating eight domains of physical and mental health.
- Gastrointestinal Quality of Life Index (GIQLI): Assessing specific digestive function (0–144 scale).

The SF-36 and GIQLI were selected in preference to endometriosis-specific instruments due to their availability in validated Romanian-language versions at the time of data collection, their established responsiveness in surgical cohorts, and their ability to enable comparison with non-endometriosis populations. The minimal clinically important difference (MCID) for SF-36 domain scores has been estimated at 5–10 points, depending on the domain, while the MCID for GIQLI has been reported at approximately 16 points on the 0–144 scale.

### 2.5. Statistical Analysis

Statistical analysis was performed using IBM SPSS Statistics for Windows, Version 28.0 (IBM Corp., Armonk, NY, USA).

Descriptive statistics were used to summarize the study cohort. Continuous variables were reported as mean ± standard deviation (SD), and categorical variables as frequencies and percentages. To evaluate changes in symptoms and quality of life over time, we conducted within-subject comparisons between the baseline measurements taken before surgery and the postoperative measurements at 6 and 12 months. This analysis was performed using paired Student’s *t*-tests and repeated-measures analysis of variance (ANOVA) for patients with complete follow-up data. All statistical tests were two-sided, and a *p*-value < 0.05 was considered statistically significant. All longitudinal comparisons were performed as complete-case analyses, limited to patients with available data at both timepoints of interest (baseline vs. 6 months; baseline vs. 12 months). Given the exploratory nature of the analyses and the use of three primary outcome domains (VAS, SF-36, GIQLI), a Holm correction for multiple comparisons was applied. Effect sizes are reported as mean differences with 95% confidence intervals. Prior to parametric testing, normality of distribution was assessed using the Shapiro–Wilk test. VAS scores, SF-36 domain scores, and GIQLI total scores demonstrated approximately normal distribution, supporting the use of paired *t*-tests for longitudinal comparisons.

## 3. Results

### 3.1. General Characteristics and Surgical Management

A total of 837 patients confirmed with endometriosis were included in this retrospective study. The mean age of the participants was 34.3 ± 6.3 years, reflecting the typical reproductive age associated with this pathology. Disease staging was performed according to the revised American Society for Reproductive Medicine (rASRM) classification and reported as stage distribution. Regarding disease severity, the majority of patients presented with advanced endometriosis: Stage IV in 450 patients (58.3%) and Stage III in 194 patients (25.1%), while early stages were less common (Stage I-II: 16%).

All surgical procedures were performed using a minimally invasive approach (primarily laparoscopy, with robotic-assisted surgery utilized in selected complex cases). The surgeries were conducted by a multidisciplinary team comprising gynecologists, general surgeons, and urologists, ensuring optimal management of extragenital involvement. The primary surgical objective was the complete macroscopic excision of all visible endometriotic lesions. Histopathological examination confirmed the diagnosis of endometriosis in all analyzed cases.

The surgical management of bowel endometriosis was tailored to the extent of the disease and the specific anatomical involvement. While 43.8% (*n* = 367) of patients underwent surgery without bowel resection, a significant proportion required colorectal interventions due to deep infiltrating endometriosis (DIE). Specifically, 39.7% (*n* = 332) underwent segmental resection, 8.1% (*n* = 68) received rectal shaving, and 1.1% (*n* = 9) underwent discoid resection. Additionally, hysterectomy was performed in 13.3% (*n* = 111) of cases as an associated procedure for definitive management of adenomyosis or other uterine pathologies.

For the longitudinal analysis of quality of life and symptom evolution, follow-up data were available for a subgroup of patients at 6 months and 12 months postoperatively. This subgroup showed similar demographic and clinical characteristics to the total cohort, ensuring that the longitudinal results are representative of the initial study population. The baseline demographic and surgical characteristics of the entire cohort are summarized in Table 1. To address the attribution rate at the 12-month follow-up, a sensitivity analysis was conducted comparing the baseline characteristics of the responders (*n* = 44) versus those lost to follow-up (*n* = 793). There were no statistically significant differences between the two groups regarding mean age (*p* = 0.77), rASRM stage distribution (*p* = 0.90), or the proportion of patients requiring bowel resection (*p* = 0.51). This indicates that the 12-month cohort is representative of the initial population and that attrition bias is unlikely to have significantly skewed the outcomes.

With regard to perioperative safety outcomes, major complications were recorded in a minority of patients undergoing segmental bowel resection. Four patients (1.2% of the segmental resection subgroup) required reoperation for anastomotic bleeding, all managed successfully without stoma formation. One case of anastomotic leak (0.3%) was identified and managed conservatively without surgical stoma. One patient (0.3%) developed a pelvic abscess requiring laparoscopic reintervention with peritoneal lavage followed by antibiotic therapy. Two patients (0.6%) developed postoperative bladder atony, resolving with conservative management. No cases of ureteral fistula or stoma formation were recorded.

### 3.2. Evolution of Pain Symptoms

**Figure 2:**
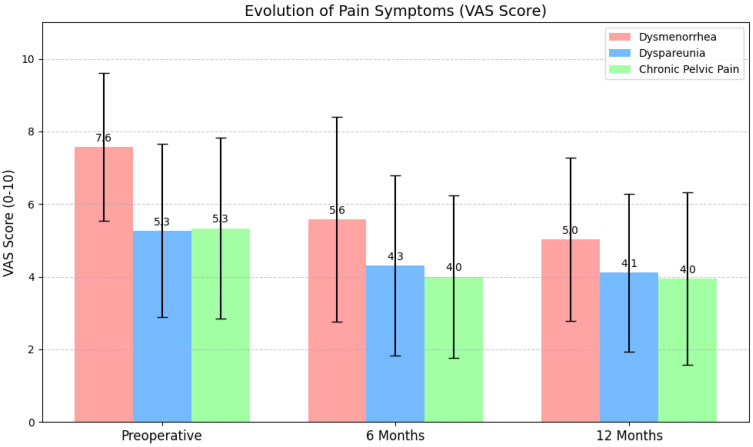
The longitudinal evolution of pain intensity for dysmenorrhea, dyspareunia, and chronic pelvic pain at baseline (prior to surgery) as well as at 6 months and 12 months following the surgical procedure, evaluated using the Visual Analog Scale (VAS). Values represent means ± standard deviation. Data were confirmed to be approximately normally distributed prior to analysis.

One of the primary objectives of the surgical treatment was the alleviation of pain symptoms, which significantly impact the quality of life in patients with endometriosis. The longitudinal evolution of pain intensity, evaluated using the Visual Analog Scale (VAS), is illustrated in Figure 2.

The analysis revealed a statistically significant reduction in pain scores across all evaluated categories at 6 months postoperatively compared to baseline. Dysmenorrhea showed the most substantial clinical improvement. As detailed in Table 2, the mean VAS score decreased significantly from a preoperative baseline of 7.6 ± 2.0 to 5.6 ± 2.8 at the 6-month follow-up (*p* < 0.001), indicating a shift from severe to moderate pain intensity. This improvement was sustained at 12 months, with a mean score of 5.0 ± 2.3 (*p* < 0.001).

Dyspareunia and chronic pelvic pain also exhibited significant improvements. The mean score for dyspareunia dropped from 5.3 ± 2.4 to 4.3 ± 2.5 at 6 months (*p* = 0.015), while chronic pelvic pain decreased from 5.3 ± 2.5 to 4.0 ± 2.2 (*p* = 0.010), as shown in Table 2. Although the statistical significance for these two parameters was not maintained in the paired analysis at 12 months, likely due to the reduced sample size in the long-term follow-up group—the absolute mean values remained lower than the preoperative baseline, suggesting a sustained clinical benefit.

### 3.3. Quality of Life Outcomes (SF-36)

**Figure 3:**
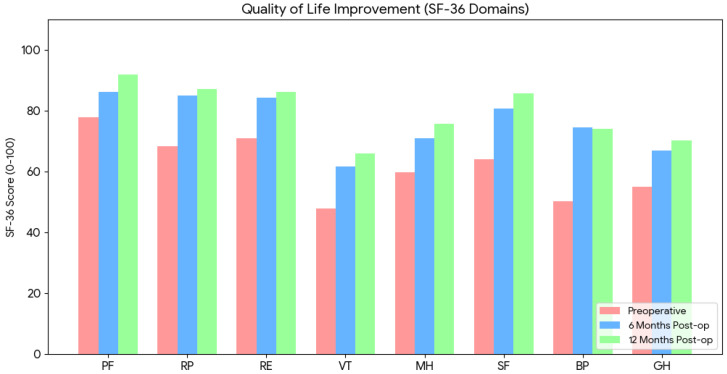
Evolution of Quality of Life scores (SF-36) across 8 domains. Higher scores indicate better health status. (Legend: PF = Physical Functioning, RP = Role Physical, RE = Role Emotional, VT = Vitality, MH = Mental Health, SF = Social Functioning, BP = Bodily Pain, GH = General Health). Values represent means ± standard deviation. Data were confirmed to be approximately normally distributed prior to analysis.

The multidimensional impact of surgical treatment on patients’ quality of life was comprehensively evaluated using the Short Form-36 (SF-36) health survey. This instrument provided a granular analysis of health status across eight distinct domains. As illustrated in Figure 3 a statistically significant improvement was observed in all dimensions at the 6-month follow-up compared to preoperative values (*p* < 0.01 for all domains), confirming the broad benefits of the intervention. The detailed evolution of mean scores is presented in Table 3.

#### 3.3.1. Physical Health Domains

The most dramatic clinical improvements were recorded in the domains related to physical well-being, which are typically the most severely affected by deep infiltrating endometriosis. The Bodily Pain (BP) domain, which serves as a proxy for the efficacy of surgical excision on dysmenorrhea and chronic pelvic pain, showed a remarkable recovery. The mean score increased by approximately 24 points, rising from a preoperative baseline of 50.2 (indicative of severe pain interference) to 74.5 at 6 months (*p* < 0.001). This upward trend stabilized at 12 months (74.0), suggesting a lasting resolution of pain symptoms.

Similarly, the Role Physical (RP) score, which assesses limitations in daily work and regular activities due to physical health, demonstrated a substantial restoration of functional capacity. The mean score surged from 68.4 preoperatively to 85.1 at 6 months (*p* < 0.001) and continued to improve to 87.1 at the 1-year mark. This shows that most patients were able to return to their work and home lives without physical limitations. Physical Functioning (PF) followed a consistent positive trajectory, reaching a near-optimal mean score of 91.9 at 12 months, significantly higher than the baseline of 77.9 (*p* < 0.001).

#### 3.3.2. Mental and Social Health Domains

Beyond the physical relief, the surgical intervention had a profound impact on the psychological burden associated with the disease. Preoperatively, patients reported low scores in Vitality (VT) (mean 47.8) and Social Functioning (SF) (mean 64.1), reflecting the fatigue and social isolation often caused by chronic pain. Postoperatively, these domains exhibited statistically significant recoveries. Social Functioning scores improved to 80.8 at 6 months and further increased to 85.6 at 12 months (*p* < 0.001), suggesting that patients successfully reintegrated into their social lives. Vitality scores increased to 65.8 at the 1-year follow-up, indicating a significant reduction in fatigue levels. The Mental Health (MH) and Role Emotional (RE) domains also showed statistically significant improvements (*p* < 0.001), highlighting the alleviation of anxiety and depressive symptoms associated with the preoperative chronic pain state.

Overall, the longitudinal data indicates that the benefits of minimally invasive excision for deep endometriosis extend beyond immediate symptom relief, offering a sustained improvement in global quality of life that persists and even continues to improve up to one year postoperatively.

### 3.4. Gastrointestinal Functional Outcomes (GIQLI)

Given the extensive bowel involvement in a significant proportion of the study population (39.7% undergoing segmental resection), the evaluation of gastrointestinal function was a critical safety endpoint. The Gastrointestinal Quality of Life Index (GIQLI) was used to quantify the cumulative impact of symptoms, physical status, and emotional well-being related to digestion.

**Figure 4:**
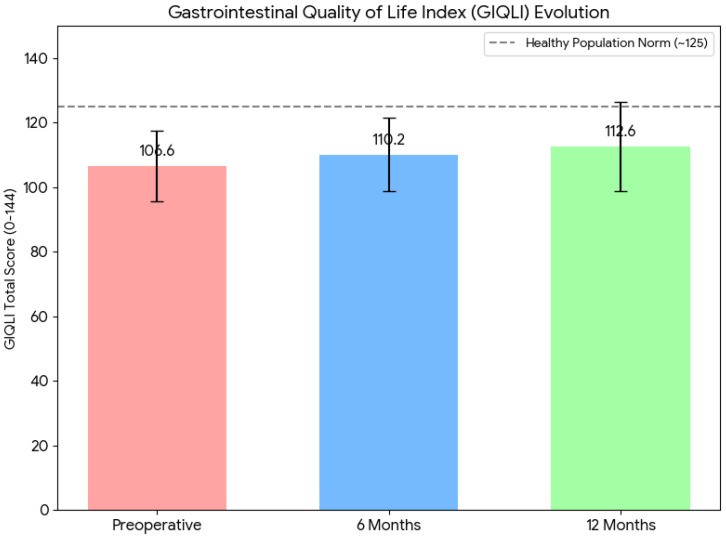
Evolution of the mean Gastrointestinal Quality of Life Index (GIQLI) total score. The dashed line represents the reference value for the healthy population (~125 points). A significant improvement is observed at 12 months.

As depicted in Figure 4, the mean total GIQLI score showed a progressive upward trend postoperatively. The mean preoperative score was 106.6 ± 11.0, which is moderately lower than the established norm for a healthy population (~125 points), reflecting the burden of catamenial digestive symptoms. At the 6-month follow-up, the score increased to 110.2 ± 11.5. While this indicated a positive trend, the statistical significance was not reached in the paired analysis (*p* = 0.335), likely reflecting the ongoing physiological adaptation and healing process of the gastrointestinal tract following extensive dissection (Table 4).

However, a statistically significant improvement was observed at the long-term follow-up. At 12 months postoperatively, the mean GIQLI score rose to 112.6 ± 13.8, significantly higher than the preoperative baseline (*p* = 0.027). A repeated-measures ANOVA was conducted to evaluate the trajectory of gastrointestinal recovery. The group-by-time interaction between the bowel resection group and the non-bowel group was not statistically significant (*p* = 0.819). This indicates that patients undergoing bowel surgery experienced parallel and comparable improvements in GIQLI scores over the 12-month follow-up compared to those without bowel involvement. The stratified outcomes at 12 months, presented in Figure 4 further illustrate this pattern: GIQLI scores were numerically higher in the segmental resection group (92.1 ± 20.5) compared to the conservative surgery group (87.5 ± 17.4), although between-group differences did not reach statistical significance (*p* = 0.078) as we can see in Table 5. Furthermore, a multivariable linear regression model was developed to predict the 12-month GIQLI outcome. The baseline pain severity (preoperative VAS) did not act as a significant confounding predictor (*p* = 0.719). The type of bowel procedure (segmental or conservative resection) emerged as the only significant independent predictor of functional improvement (*p* < 0.01), maintaining its significance even when adjusting for patient age and baseline disease severity. This finding suggests that while immediate functional recovery may be gradual, the long-term benefit of surgical excision is robust, with patients achieving a functional status superior to their preoperative condition without developing de novo severe dysfunction.

**Figure 5:**
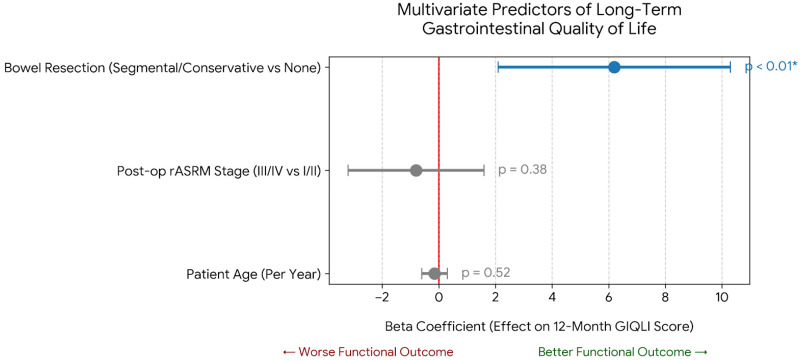
Forest plot illustrating the multivariable linear regression analysis for predictors of long-term gastrointestinal quality of life (GIQLI score at 12 months). The type of bowel procedure emerged as the only significant independent predictor of functional improvement (*p* < 0.01), after adjustment for patient age and postoperative disease severity (rASRM stage). * *p* < 0.05, statistically significant.

Figure 5 displays three covariates along the *y*-axis: bowel resection (segmental/conservative vs. none), postoperative rASRM stage (III/IV vs. I/II), and patient age (per year). The *x*-axis represents the estimated beta coefficients, indicating the direction and magnitude of each variable’s effect on functional outcome. Positive coefficients (to the right of zero) indicate improved gastrointestinal quality of life, while negative coefficients (to the left) suggest worse outcomes. A vertical red line at zero denotes no effect. Bowel resection shows a strong positive association with improved long-term GIQLI scores, with a large effect size and a statistically significant *p*-value (*p* < 0.01). In contrast, postoperative rASRM stage demonstrates a negative but non-significant association (*p* = 0.38), indicating no clear evidence of an independent effect on postoperative quality of life. Similarly, patient age has a negligible effect size and is not statistically significant (*p* = 0.52). Overall, the analysis suggests that bowel resection is the only independent predictor significantly associated with improved long-term gastrointestinal quality of life in this model, while surgical disease stage and age do not appear to have a meaningful impact after adjustment.

## 4. Discussion

The present multicenter study, conducted on a large cohort of 837 patients, confirms that minimally invasive excision of endometriosis significantly improves health-related quality of life (HRQoL) and provides sustained pain relief. Our findings are consistent with the growing body of evidence supporting the “radical but nerve-sparing” surgical philosophy, particularly in cases of deep infiltrating endometriosis (DIE) with colorectal involvement.

### 4.1. Impact on Pain Symptoms

The primary indication for surgery in our cohort was pain refractory to medical treatment. We observed a substantial and statistically significant reduction in VAS scores for dysmenorrhea (from 7.6 to 5.0) and chronic pelvic pain at the 12-month follow-up. These results align with those reported by Rindos et al. [1], who demonstrated significant improvements in pain and quality of life following laparoscopic excision of endometriosis. Furthermore, the significant decrease in dyspareunia (*p* = 0.015 at 6 months) supports the findings of Coroleucă et al. [5], suggesting that the excision of retrocervical and uterosacral nodules restores sexual function and, implicitly, the intimate dimension of quality of life. Recent qualitative studies have further emphasized that beyond the measurable pain reduction, patients experience profound improvements in their emotional well-being and ability to engage in social relationships, dimensions that are often overlooked in purely quantitative assessments [2,4]. The reduction in dysmenorrhea was not only statistically significant but also clinically meaningful, as the absolute decrease in the VAS score exceeds the Minimal Clinically Important Difference (MCID) threshold. While the reductions in dyspareunia and chronic pelvic pain lost statistical significance at 12 months, this is most likely attributable to a Type II statistical error secondary to the significantly reduced sample size, rather than a true clinical recurrence. This multifaceted impact underscores the importance of comprehensive outcome evaluation that extends beyond pain scores alone.

### 4.2. Quality of Life Recovery (SF-36)

Beyond simple pain control, our study utilized the SF-36 survey to map the multidimensional recovery of patients. Preoperatively, our cohort presented low scores in Vitality and Social Functioning, reflecting the “burden of disease” described in the recent review by Manu et al. [11]. Postoperatively, we registered a dramatic improvement in the Role Physical domain (from 68.4 to 87.1), indicating that patients could return to their professional and daily activities without physical limitations. These findings are comparable to the large cohort results published by Dubernard et al. [4], who reported similar trajectories in physical component scores following colorectal endometriosis surgery. The SF-36 has been extensively validated in endometriosis populations and demonstrates excellent responsiveness to treatment effects, particularly in physical functioning and bodily pain domains, making it an ideal instrument for capturing the breadth of surgical impact [10,14].

However, unlike some studies, where mental health scores showed slower recovery, our data indicates a significant improvement in Emotional Role and Mental Health as early as 6 months [20]. This rapid psychological recovery may be attributed to the multidisciplinary preoperative counseling and the definitive nature of the excision, which likely reduces patient anxiety regarding disease progression. Nevertheless, longitudinal studies have revealed that recovery trajectories are not uniform across all patients [20,22]. Comptour et al. [22] identified distinct subgroups with different patterns of quality of life evolution, with some patients experiencing immediate and sustained improvement while others show delayed or incomplete recovery. Predictive factors for optimal outcomes include younger age, complete excision of visible lesions, and lower baseline pain intensity [23], suggesting that individualized prognostic counseling based on patient characteristics may enhance realistic expectation setting and postoperative satisfaction.

### 4.3. Gastrointestinal Function (GIQLI)

A central concern in endometriosis surgery, particularly when segmental resection is performed (39.7% of our cases), is the risk of postoperative bowel dysfunction. Our analysis of the GIQLI scores provides reassuring data. Although the improvement was not statistically significant at 6 months (reflecting the physiological healing period), the score significantly increased at 12 months (112.6 vs. 106.6 baseline, *p* = 0.027). It should be noted that the observed mean improvement at 12 months (+6.0 points) falls below the established minimal clinically important difference (MCID) for GIQLI of approximately 16 points, suggesting that while statistically significant, the change may not reach the threshold of clinical meaningfulness at the group level. This finding should be interpreted with caution and warrants confirmation in larger prospective cohorts with complete follow-up. This “U-shaped” recovery curve mirrors the observations and has been confirmed in recent prospective long-term evaluations demonstrating progressive improvement in bowel function, with initial transient deterioration in some parameters resolving by 12 months [21,28,32].

Our results contradict the historical apprehension that radical bowel surgery leads to permanent functional impairment. On the contrary, removing the stenotic or irritative nodule improves intestinal quality of life, a conclusion also supported by Ianieri et al. [26] in the context of nerve-sparing techniques. Recent systematic reviews and meta-analyses have provided important context for surgical decision-making in bowel endometriosis. Darici et al. [27] demonstrated in their comprehensive meta-analysis that while segmental resection achieves superior pain relief, it is associated with higher rates of certain bowel complaints compared to conservative approaches such as shaving or discoid resection. However, these bowel symptoms tend to be transient, and long-term functional outcomes remain favorable [26]. The meta-analysis by Maguire et al. [33] specifically focusing on colorectal endometriosis confirmed robust improvements in both generic (SF-36) and disease-specific (EHP-30) QoL measures at 6 and 12 months postoperatively, with effect sizes most pronounced in the physical health domains, thus corroborating our findings.

### 4.4. Clinical and Molecular Implications

The complexity of the cases in our study, with a high prevalence of severe DIE, underscores the necessity of high-volume centers. As highlighted by Coroleucă et al. [5], the aggressive behavior of endometriosis is dictated by its molecular profile (Ki-67, Bcl-2). Therefore, complete excision remains the only modality to address the biological activity of the lesion locally. Our high rate of histopathological confirmation and the significant long-term symptom relief validate the accuracy of the preoperative imaging protocol (TVUS/MRI) utilized in our centers, consistent with Baușic et al. [8]. The integration of molecular profiling with clinical and imaging data may in the future allow for more personalized surgical planning and prognostication, though this remains an area requiring further investigation.

### 4.5. Strengths and Limitations

The main strengths of this study include the large sample size (N = 837), the multicenter design, and the use of a standardized, multimodal PROM set (SF-36 + GIQLI). The systematic use of validated PROMs aligns with contemporary recommendations emphasizing the importance of patient-reported outcomes in capturing the full spectrum of disease impact [11,13]. To our knowledge, this is one of the largest cohorts in Eastern Europe evaluating functional outcomes after DIE surgery.

However, several limitations must be acknowledged. First, the retrospective design and the attrition rate at the 12-month follow-up (a common issue in longitudinal studies, as noted by Szabo et al. [29]) may introduce selection bias. It is possible that patients with excellent recovery were less motivated to return for follow-up compared to symptomatic ones, or conversely, that unsatisfied patients sought care elsewhere. Nevertheless, the baseline characteristics of the responder subgroup were similar to the total cohort, minimizing this bias. Second, we did not stratify GIQLI scores strictly by the type of bowel procedure (shaving vs. resection) in the longitudinal analysis due to the smaller sample size of the subgroups, which remains a subject for future prospective research. Another limitation is the incomplete documentation regarding long-term adherence to postoperative hormonal suppression. Because patients were followed up across various territorial centers, incorporating medical therapy into the multivariable regression model would have critically reduced the sample size; thus, the independent effect of postoperative medical suppression could not be fully isolated. Future studies employing trajectory analysis methodologies similar to those used by Comptour et al. [22] may help identify preoperative predictors of different recovery patterns, thereby facilitating more tailored patient counseling.

## 5. Conclusions

Minimally invasive surgical excision of endometriosis, performed in a high-volume multidisciplinary setting, represents a robust therapeutic strategy that offers significant and sustained improvements in health-related quality of life. Our study confirms that the complete removal of endometriotic lesions effectively breaks the cycle of chronic pain, allowing patients to regain their physical and social functionality as early as 6 months postoperatively.

Crucially, our findings dispel the apprehension surrounding bowel surgery; we demonstrated that even in cases requiring segmental resection, gastrointestinal function is not permanently compromised. On the contrary, digestive quality of life scores (GIQLI) show a significant improvement at 1 year compared to the preoperative baseline, highlighting the long-term benefits of removing the obstructive or irritative pathology. We advocate for the routine integration of standardized patient-reported outcome measures such as SF-36 and GIQLI in clinical practice, as they provide an objective, patient-centered metric of surgical success that extends beyond simple anatomical recurrence rates.

## Figures and Tables

**Table 1:** The demographic and surgical characteristics summarization of the cohort (*n* = 837).

Variable	Total Cohort (*n* = 837)
Age (years), mean ± SD	34.3 ± 6.3
Surgical Procedure, *n* (%)	
No Bowel Resection	367 (43.8%)
Rectal Shaving	68 (8.1%)
Discoid Resection	9 (1.1%)
Segmental Resection	332 (39.7%)

**Table 2:** Changes in VAS scores over time (mean ± SD) with *p*-values from paired *t*-test analysis provided, considering a 0.05 significance level. Paired comparisons: Dysmenorrhea baseline vs. 6 months *n* = 65, vs. 12 months *n* = 27; Dyspareunia baseline vs. 6 months *n* = 37, vs. 12 months *n* = 15; Chronic Pelvic Pain baseline vs. 6 months *n* = 37, vs. 12 months *n* = 14. * *p* < 0.05, statistically significant.

Symptom	Preoperative (Mean ± SD)	6 Months Post-Op (Mean ± SD)	Mean Difference (95% CI)	*p*-Value	12 Months Post-Op (Mean ± SD)	Mean Difference (95% CI)	*p*-Value
Dysmenorrhea	7.6 ± 2.0 (*n* = 233)	5.6 ± 2.8 (*n* = 69)	−2.46 (−3.24 to −1.69)	<0.001 *	5.0 ± 2.3 (*n* = 30)	−2.70 (−3.88 to −1.53)	<0.001 *
Dyspareunia	5.3 ± 2.4 (*n* = 167)	4.3 ± 2.5 (*n* = 42)	−1.08 (−1.92 to −0.24)	0.015 *	4.1 ± 2.2 (*n* = 18)	−0.73 (−2.32 to 0.85)	0.379
Chronic Pelvic Pain	5.3 ± 2.5 (*n* = 197)	4.0 ± 2.2 (*n* = 42)	−1.30 (−2.24 to −0.36)	0.010 *	4.0 ± 2.4 (*n* = 20)	−0.64 (−2.28 to 1.00)	0.455

**Table 3:** Changes in Quality of Life mean scores (SF-36) over time, with *p*-values from paired *t*-test analysis provided, considering a 0.05 significance level. Paired comparisons: baseline vs. 6 months *n* = 99; baseline vs. 12 months *n* = 45. * *p* < 0.05, statistically significant.

SF-36 Domain	Preoperative (*n* = 261) (Mean)	6 Months Post-Op (*n* = 100) (Mean)	Mean Difference (95% CI)	*p*-Value	12 Months Post-Op (*n* = 46) (Mean)	Mean Difference (95% CI)	*p*-Value
Physical Functioning (PF)	77.9 ± 25.9	86.3 ± 18.4	+8.4 (3.9 to 12.9)	0.009 *	91.9 ± 12.1	+14.0 (7.5 to 20.5)	<0.001 *
Role Physical (RP)	68.4 ± 28.5	85.1 ± 19.3	+16.7 (11.8 to 21.6)	<0.001 *	87.1 ± 16.2	+18.7 (11.5 to 25.9)	0.001 *
Bodily Pain (BP)	50.2 ± 21.8	74.5 ± 18.5	+24.3 (20.3 to 28.3)	<0.001 *	74.0 ± 19.1	+23.8 (17.9 to 29.7)	<0.001 *
General Health (GH)	55.0 ± 22.6	66.8 ± 19.2	+11.8 (7.7 to 15.9)	<0.001 *	70.3 ± 18.8	+15.3 (9.2 to 21.4)	0.001 *
Vitality (VT)	47.8 ± 20.1	61.7 ± 18.4	+13.9 (10.1 to 17.7)	<0.001 *	65.8 ± 17.9	+18.0 (12.5 to 23.5)	<0.001 *
Social Functioning (SF)	64.1 ± 24.3	80.8 ± 18.1	+16.7 (12.4 to 21.0)	<0.001 *	85.6 ± 15.5	+21.5 (15.3 to 27.7)	<0.001 *
Role Emotional (RE)	71.0 ± 26.8	84.2 ± 19.9	+13.2 (8.5 to 17.9)	<0.001 *	86.2 ± 17.6	+15.2 (8.4 to 22.0)	<0.001 *
Mental Health (MH)	59.9 ± 21.4	70.9 ± 18.6	+11.0 (7.1 to 14.9)	<0.001 *	75.8 ± 16.4	+15.9 (10.3 to 21.5)	<0.001 *

**Table 4:** Changes in the total GIQLI mean score over time, ranging from 0 to 144, with *p*-values from paired *t*-test analysis provided for comparisons between 6 or 12 months and preoperative time, considering a significance level of 0.05. * *p* < 0.05, statistically significant.

Time Point	Mean Score ± SD	Range (Min–Max)	Mean Difference	*p*-Value (vs. Pre-Op)
Preoperative (*n* = 262)	106.6 ± 11.0	81.3–132.4		-
6 Months Post-op (*n* = 98)	110.2 ± 11.5	79.2–132.4	+3.6 (1.4 to 5.8)	0.335
12 Months Post-op (*n* = 44)	112.6 ± 13.8	72.0–133.1	+6.0 (2.3 to 9.7)	0.027 *

**Table 5:** Functional and pain outcomes at 12 months stratified by surgical procedure. Baseline GIQLI scores were comparable across surgical subgroups (No Bowel Resection: 110.8 ± 22.4; Conservative Surgery: 103.4 ± 27.9; Segmental Resection: 103.4 ± 23.7). Stratified 12-month outcomes for the conservative surgery subgroup are not reported separately due to limited follow-up data (*n* = 3).

Variable at 12 Months Follow-Up	No Bowel Resection	Conservative Bowel Surgery (Shaving/Discoid)	Segmental Bowel Resection	*p*-Value (Between Groups)
VAS Dysmenorrhea (Mean ± SD)	7.9 ± 2.0	8.9 ± 1.2	8.2 ± 2.0	0.121
GIQLI Score (Mean ± SD)	104.6 ± 14.2	87.5 ± 17.4	92.1 ± 20.5	0.078

## Data Availability

The data are available from the corresponding author upon reasonable request.

## Abbreviations

The following abbreviations are used in this manuscript:

DIE	Deep Infiltrating Endometriosis
HRQoL	Health-Related Quality Of Life
VAS	Visual Analog Scale
SF-36	Short Form-36
GIQLI	Gastrointestinal Quality of Life Index
PROMs	Patient-Reported Outcome Measures
ER	Estrogen Receptors
PR	Progesterone Receptors
TVUS	Transvaginal Ultrasound
MRI	Magnetic Resonance Imaging
EHP-30	Endometriosis Health Profile
MIS	Minimally invasive surgery
QoL	Quality of Life
IBD	Inflammatory Bowel Disease
GnRH	Gonadotropin-Releasing Hormone
SD	Standard Deviation
PF	Physical Functioning
RP	Role Physical
BP	Bodily Pain
GH	General Health
VT	Vitality
SF	Social Functioning
RE	Role Emotional
MH	Mental Health
ESD	Endometriosis Symptom Diary
EIS	Endometriosis Impact Scale
